# A Lightweight Plant Disease Detection Model for Long-Tailed Agricultural Scenarios

**DOI:** 10.3390/plants15081206

**Published:** 2026-04-15

**Authors:** Luyun Chen, Yuzhu Wu, Yangyuzhi Meng, Qiang Tang, Zhen Tian, Shengyu Li, Siyuan Liu

**Affiliations:** 1 School of Information Science and Engineering, Northeastern University, Shenyang 110819, China; chenly2@mails.neu.edu.cn (L.C.); wy2040979673@126.com (S.L.); 2 College of Engineering, Northeast Agricultural University, Harbin 150030, China; a07220052@neau.edu.cn; 3 College of Electrical and Information Engineering, Northeast Agricultural University, Harbin 150030, China; a19240036@neau.edu.cn; 4 School of Artificial Intelligence, Anhui University of Science and Technology, Hefei 231131, China; 5 James Watt School of Engineering, University of Glasgow, Glasgow G12 8QQ, UK; 2620920z@student.gla.ac.uk; 6 School of Civil and Hydraulic Engineering, Chongqing University of Science and Technology, Chongqing 401331, China; lishengyu.mr@gmail.com

**Keywords:** plant disease detection, long-tailed distribution, lightweight object detection, data augmentation, model pruning, knowledge distillation, precision agriculture

## Abstract

In natural agricultural environments, plant disease monitoring faces significant challenges, including a highly uneven (long-tail) distribution of disease species, tiny scales of early-stage lesions, and complex, variable backgrounds. These factors hinder the ability of existing lightweight models to balance detection accuracy and computational efficiency. To address these issues, this paper proposes a detection scheme driven by the synergy of data distribution reshaping and model architecture optimization. At the data level, we propose the CALM-Aug augmentation strategy. Based on the statistical distribution characteristics of disease categories, this strategy utilizes object-level copy-paste logic to specifically compensate for the feature shortcomings of rare disease samples. It introduces a teacher-guided screening mechanism and employs accept–reject sampling to ensure the pathological consistency of the augmented samples, thereby alleviating the model’s inductive bias toward head categories. At the model architecture level, using YOLOv11 as the baseline, the YOLO11-ARL model adapted to agricultural scenarios is constructed. It enhances sensitivity to early point-like disease spots through Efficient Multi-Scale Convolutional Pyramids and lightweight decoupled detection heads. Furthermore, a Layer-wise Adaptive Feature-guided Distillation Pruning (LAFDP) algorithm is utilized to extract a lightweight version, YOLO11-ARL-PD, achieving a significant reduction in parameters and computational cost. Experimental results on the PlantDoc dataset show that the final model achieves a precision of 89.0% and an mAP@0.5 of 85.3%. Compared to the baseline model YOLOv11n, YOLO11-ARL-PD improves precision and average precision by 7.7 and 2.6 percentage points, respectively, while reducing parameters by 51.93% and weights by 46.15%. Cross-dataset tests prove the good generalization performance of the proposed method. This study indicates that, under lightweight constraints, jointly optimizing the training distribution and model architecture is an effective way to improve plant disease monitoring and to support the edge deployment of smart crop-protection systems. All resources for CALM-Aug are available at wyz-2004/CALM-Aug on GitHub.

## 1. Introduction

Plant disease detection is a fundamental issue in precision agriculture, yet the complexity of its practical application is often underestimated. Compared to general object detection, field images exhibit more pronounced class imbalance, a higher proportion of small-sized objects, and relatively complex background interference. Furthermore, detection models must perform real-time inference operations within limited computational capabilities while ensuring stable and accurate classification even under adverse conditions such as weak texture or partial occlusion. When these “high constraints combined with high complexity” intertwine, many lightweight models that perform excellently on standard datasets struggle to replicate that level of performance in real agricultural environments.

Current research on plant disease detection primarily focuses on data augmentation and network optimization. While the former has alleviated the issue of insufficient absolute quantities of disease images to some extent, the “long-tail distribution” phenomenon—where common diseases recur frequently while rare ones are rarely observed—remains unaddressed for data collected in actual field settings. Abdullah et al. utilized InstaGAN to augment disease images, but its generalizability is limited by the scale of the categories [1]. The method proposed by Wassim Benabbas et al. still leaves room for improvement in complex-background scenarios [2]. In recent years, the target-level copy-paste strategy has demonstrated strong robustness in general object detection because it can break contextual constraints and directly increase the representation of tail categories. However, directly applying traditional copy-paste to agricultural scenarios has obvious limitations: first, the lack of pathological logical consistency means that blind, random pasting may generate noise-like samples that violate biological common sense, such as lesions appearing in non-host regions; second, simple overlapping easily introduces boundary artifacts, causing the model to overfit to artificially synthesized traces.

Another category of research focuses on enhancing network architecture, such as introducing multi-scale feature branches, attention mechanisms, or more complex feature fusion modules, to improve the model’s sensitivity to lesion details. Jie Ding et al. utilized the RFCA ResNet model; however, while their data augmentation strategy improved accuracy, it came at the cost of some precision [3]. Zhengjie Ji et al., based their work on ResNet50, adopted a Deep Separable Residual (DSR) structure; however, to achieve model lightweighting, the introduction of DSR led to a decrease in accuracy [4]. Thus, while these methods can indeed enhance feature representation, they are often accompanied by a surge in the number of parameters and increased computational burden, which conflicts with the lightweighting requirements of edge devices such as drones and mobile terminals in agricultural scenarios. Furthermore, traditional pruning methods typically employ a uniform global pruning ratio, which tends to overlook the distribution differences of lesion features across different layers, leading to the erroneous removal of edge texture features from small lesions. Additionally, relying on a single evaluation metric—such as weight values alone—makes it difficult to accurately identify the critical paths that contribute significantly to pathological features.

Essentially, there is a fundamental mismatch between the non-uniformity of field data, the expression pathways of lesion features, and the capacity constraints of lightweight models. The faint features of rare diseases are easily masked by high-frequency signals, and the tasks of precisely locating and classifying early-stage, small lesions face gradient interference within the parameter space.

Yang X et al. enhanced the PlantDoc dataset by modifying the YOLOv10n model and introducing the DSConv module, achieving high detection efficiency and model compactness with an mAP50 of 65.4%, while significantly reducing the number of parameters [5]. Sun Hao et al. addressed the challenges of leaf occlusion and small lesion areas in tomato leaf disease detection by proposing the Efficient Tomato Disease Detection Network (E-TomatoDet). This network enhances global feature capture to learn and integrate multi-scale local features, achieving an mAP50 of 51.8% on the PlantDoc dataset, outperforming YOLOv10s [6].

Building on such research, this paper focuses on reorganizing effective supervision signals and key pathological expression pathways. At the data level, the training distribution is reshaped through a category-aware lesion augmentation mechanism (CALM-Aug). This mechanism utilizes lesion-level reconstruction combined with a teacher model for filtering, ensuring that rare lesions are fully covered across diverse backgrounds and scales. Compared to traditional copy-paste methods, CALM-Aug employs “accept-reject sampling” to ensure that augmented samples align with both pathological logic and visual distributions. This addresses the issue of noise-introduced supervision inherent in traditional copy-paste methods at the source, achieving a more targeted adjustment of the empirical distribution than conventional augmentation.

At the architectural level, this paper focuses on refining the network at gradient-sensitive locations. We introduce Efficient Multi-Scale Convolution Units (EMSCP) to capture lesion features ranging from punctate to patchy patterns through parallel multi-receptive-field branches; we also design a Lightweight Decoupled Detection Head (LADH), which reduces parameter sharing and introduces a dynamically weighted loss function to enable smoother boundary learning for small lesions while maintaining network topological stability.

To satisfy the real-time deployment requirements of agricultural inspection terminals, we further introduce the Layer-wise Adaptive Feature-Guided Distillation Pruning (LAFDP) algorithm. LAFDP adopts a two-stage compression strategy that combines structured pruning with distillation-based recovery. In the first stage, redundant channels are structurally pruned under a predefined compression target, thereby reducing both the parameter count and the computational burden. In the second stage, the unpruned model serves as the teacher, while the pruned model is fine-tuned via knowledge distillation to restore its discriminative capacity at the levels of both output distributions and intermediate feature representations. Through this continuous compression-and-recovery framework, the model becomes significantly lighter while still preserving stable recognition performance for small lesions in complex agricultural backgrounds.

Experimental results indicate that the performance improvement stems from the reshaping of the underlying data distribution and the reorganization of computational paths, rather than volume expansion. Such systematic adjustments under lightweight constraints hold significant practical implications for intelligent crop protection scenarios with complex environments.

## 2. Materials and Methods

### 2.1. Dataset Description

This study utilizes the publicly available plant disease detection dataset PlantDoc to establish the experimental framework. The PlantDoc dataset originates from real-world field environments and includes images of plant leaves under natural lighting, in complex backgrounds, with occlusion phenomena, and at different growth stages, enabling it to accurately reflect real-world detection conditions in agricultural production scenarios [7].

The dataset comprises 30 classes covering various crops and common disease types, including healthy and diseased leaves of apples, tomatoes, corn, grapes, potatoes, strawberries, cherries, soybeans, and other crops. All images in the dataset are annotated in the standard YOLO format, with each annotation file recording the class index and normalized bounding box coordinates (x, y, w, h). The dataset has the following characteristics:

1. A large number of classes (30), with complex visual differences between them.
2. Diverse background environments, including natural lighting, shadows, and complex textured backgrounds.
3. Significant variation in target scale, ranging from small early-stage lesions to large-scale, widespread diseases.
4. Significant class imbalance.

To ensure experimental fairness and the reliability of model evaluation, the dataset is split into training, validation, and test sets in an 8:1:1 ratio. Specifically, 80% of the data is used to train model parameters independently, 10% is used for model debugging and selecting early stopping strategies, and the remaining 10% serves as an independent test set for final model performance evaluation [8]. The dataset is partitioned using stratified sampling to ensure that the distribution of each class across the three subsets is roughly equal.

However, statistical analysis indicates that there are significant differences in the number of instances across categories in the training set $N_{i}$. Let the category distribution vector be the following:

$$N=\left(N_{1},N_{2},\ldots ,N_{30}\right),$$

Then, the class imbalance satisfies the following:

$$\underset{i}{max}\;N_{i}\gg \underset{i}{min}\;N_{i}$$

This long-tail distribution indicates that when performing empirical risk minimization, gradient updates are largely dominated by high-frequency classes, thereby weakening the model’s learning effectiveness for rare diseases. Furthermore, the distribution in the target space tends to cluster in the middle range, with few samples of small-sized lesions, which increases the difficulty of identification [9].

**Figure 1:**
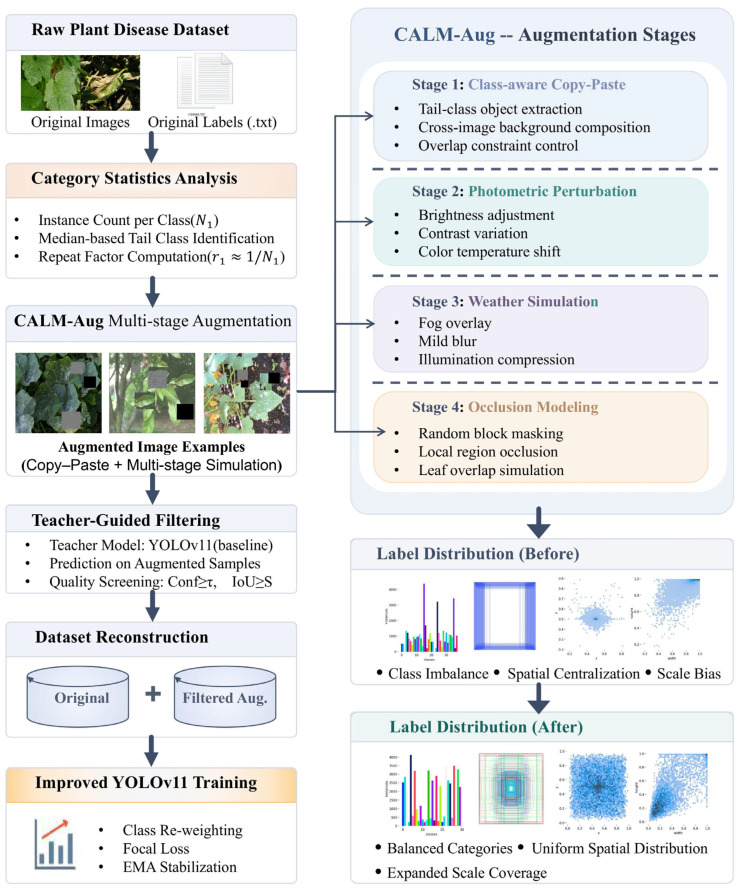
Data processing pipeline of the proposed CALM-Aug framework.

The statistical characteristics of the class and spatial distributions prior to augmentation are shown in Figure 1 (before augmentation). It is evident that issues such as class imbalance, spatial clustering, and lack of scale coverage exist. Since these statistical properties directly affect the model’s generalization ability, structural reshaping of the data distribution is necessary before training.

Based on the above analysis, this paper proposes the CALM-Aug data layer improvement architecture, which reshapes the training data according to class recognition to mitigate long-tail bias and improve the model’s adaptability to complex field scenarios.

### 2.2. CALM-Aug Framework

The PlantDoc dataset, collected under real-world field conditions, inherently contains interfering factors such as class frequency disparities, complex backgrounds, occlusions, and scale variations. Such data often exhibits a composite statistical structure characterized by “long-tail classes, spatial central bias, and insufficient scale coverage” [10]. Under this structure, a small number of high-frequency head categories continuously provide a large amount of gradient signals, while tail category samples are diluted by high-frequency signals during the lengthy training cycle; furthermore, due to shooting habits, targets are often overly concentrated in the central regions of images, resulting in extremely weak generalization capabilities of the model for edge regions. Furthermore, since early lesions often manifest as small-scale, low-contrast, subtle textural changes, their effective supervisory signals are easily overwhelmed by higher-level abstract semantics during the learning process of multi-scale networks. Therefore, the design goal of CALM-Aug is not merely to “increase the amount of data”, but to elevate the augmentation process to a form of distribution reconstruction. Its objective is to comprehensively adjust the statistical properties of the training data across three dimensions—category, space, and scale—without introducing uncontrollable noise. This approach guides gradient descent toward minimizing training risk in a manner that better matches the risk reduction required in real agricultural scenarios.

The overall workflow of CALM-Aug is shown in Figure 1.

Figure 1 comprehensively illustrates the processing workflow of CALM-Aug, ranging from raw data statistics, category-aware augmentation, multi-stage environment simulation, to teacher selection and data reconstruction. The label distribution comparison results on the right side of the figure clearly reflect the differences across three dimensions—category distribution, target center distribution, and scale distribution—before and after augmentation: prior to augmentation, there is a pronounced long-tail phenomenon and a tendency toward spatial concentration, whereas after augmentation, categories are more balanced, target spatial locations are more dispersed, and the scale coverage is broader.

#### 2.2.1. From Tail-Class Dilution to Effective Gradients: Improving Generalization Through Debiasing

Due to the vast differences in the occurrence frequencies of various disease types, the model is prone to developing an inductive bias toward common diseases during training. During object detection training, category-related supervision primarily relies on classification loss and its gradients to drive parameter updates. If the frequency of categories in the training set varies significantly, then during each mini-batch update, the majority of samples come from the head categories. The optimizer effectively operates on a biased category prior, continuously prioritizing a reduction in errors for common diseases, while tail-end diseases, due to their limited participation in updates, struggle to have their pathological features fully learned by the model. The core principle of the first layer of CALM-Aug is to adjust the frequency of training samples through category-aware augmentation, thereby altering the composition of the gradient expectations.

The key point is that CALM-Aug does not simply replicate the entire image; instead, it uses lesion-level copy-paste to embed tail-category targets into diverse backgrounds, artificially increasing the appearance frequency of rare lesions in mini-batch training. This ensures the model can obtain sufficient gradient information to characterize these rare pathological features. More importantly, this copy-paste process does not merely replicate the original samples; instead, it places the same lesion in diverse natural backgrounds, effectively expanding the limited real-world samples into a large number of “equivalent supervised events”. The combination of lesions with different backgrounds frees the model from the constraints of the original scene during the learning process, avoiding the risk of overfitting caused by sparse samples. Through this “effective gradient redistribution” at the physical level, rare diseases can be repeatedly identified by the model in more diverse scenarios that closely resemble real-world field conditions, ultimately significantly improving the detection accuracy of various diseases under a long-tail distribution.

Second, this framework demonstrates exceptional spatial debiasing capabilities, successfully transforming the center-of-mass prior from a model-exploited “shortcut” into a truly generalizable feature recognition capability. Agricultural data collection often carries an implicit bias due to the photographer’s habits: disease lesions are almost always centered in the frame, resulting in a highly concentrated distribution of targets in the training data. When trained on such data, detection models easily learn a “shortcut”—assuming that targets are more likely to appear in the central region—and thus tend to generate candidate bounding boxes more frequently in the center while being more conservative in the peripheral areas. While this shortcut may not significantly lower metrics across the entire training set, it can lead to missed detections at the edges in practical applications—such as drone inspections, casual mobile photography, or scenarios involving occluded or offset targets.

CALM-Aug utilizes object-level cross-image pasting technology to naturally introduce a spatial redistribution mechanism. Rare-class targets are randomly and uniformly distributed across all corners of the frame, particularly in the edge regions that are extremely sparse in the original data, thereby forcibly expanding the support domain of the target’s central position. Combined with multi-stage physical perturbations such as cropping, scaling, and occlusion, the model is forced to learn “target-background separability” across more spatial positions during training, thereby weakening the influence of the central bias. The change in center distribution shown on the right side of Figure 1 illustrates this: after enhancement, the coverage of target positions becomes more uniform, and the centralization of centers is alleviated. The essence of this mechanism lies in completely eliminating the negative impact of center priors, allowing the classifier to rely on transferable local textures and contextual logic.

#### 2.2.2. Scale and Visibility Remodeling and Teacher-Assisted Training

Early-stage lesion targets are often characterized by small scale, blurred boundaries, and extremely low contrast. Consequently, even with meticulous annotation, their supervisory signals are easily lost during the forward propagation of the detection network, falling into a “low-gradient region” that produces weak or unstable backpropagation gradients. Based on this, CALM-Aug’s multi-stage augmentation acts simultaneously on both scale and visibility:

On one hand, it forcibly expands the scale distribution boundaries of targets through a combination of copy-paste and random geometric scaling, ensuring that small-scale lesions are no longer confined to the morphologies of a few original samples; on the other hand, it innovatively incorporates severe luminance perturbations, simulated adverse weather conditions, and complex physical occlusion modeling, directly introducing “visibility degradation” into the training stream. This forces the model to learn how to maintain recognizability under conditions of low contrast, partial occlusion, and slight blurring. For detection tasks, this effectively extends the training process from “ideal visibility conditions” to a “difficult sample manifold” that more closely mirrors real-world deployment scenarios, thereby significantly enhancing the model’s robustness. The changes in scale coverage and the broader distribution after augmentation shown on the right side of Figure 1 are a direct illustration of this mechanism.

Simultaneously, to ensure sample quality, CALM-Aug refines the teacher-guided filtering mechanism into a rigorous “accept-reject sampling” process. Objectively speaking, any data augmentation method inevitably introduces out-of-distribution risks: when issues such as unreasonable pasting positions, jarring or incongruous local appearances, excessive physical occlusion, or inconsistent bounding box semantics arise, augmented samples may become “supervised noise”, increasing the network’s training error. In response to this, the teacher-guided filtering mechanism adopted by CALM-Aug should not be simply viewed as a post-processing step, but should be strictly understood as an accept–reject sampling process. Specifically, based on the initial distribution $\tilde{\;p}(x,y)$, it relies on the teacher model’s confidence scores and localization consistency to filter out certain samples, yielding a quality-controlled augmented distribution $\;{\tilde{p}}_{\mathrm{keep}}(x,y)$. Intuitively, teacher screening effectively trims the “noisy tail” of the augmented samples, ensuring that the performance gains from data augmentation primarily stem from samples with high consistency, rather than being overwhelmed by a small number of extreme outliers.

From the perspective of training stability, this mechanism essentially imposes an effective constraint on the additional variance introduced by augmentation operations: during the process of expanding sample diversity, the perturbations in the gradient direction caused by “incorrect augmentation” are kept within a reasonable range. This allows CALM-Aug to maintain good training convergence and experimental reproducibility even when expanding the supported range of categories, spaces, and dimensions.

#### 2.2.3. Performance Validation

To evaluate the independent contribution of CALM-Aug at the data level, we compare the detection performance of the model under different augmentation methods while keeping the network architecture, training parameters, and data split completely consistent, and only replacing the data augmentation strategy used during the training phase. Except for the augmentation method, all other training settings remain unchanged. Table 1 presents a comparison of results for the no augmentation, standard copy-paste, category-aware copy-paste, CALM-Aug variant without the teacher filtering mechanism, and full CALM-Aug approaches.

Without altering the network architecture, different augmentation strategies all yielded varying degrees of performance improvements, with the full CALM-Aug performing best across all four metrics. In particular, the simultaneous improvement in mAP50 and mAP50–95 indicates that the benefits of this method are not limited to samples that are easier to detect under loose IoU thresholds, but can also consistently improve the model’s detection performance under stricter matching criteria.

Performance differences are closely linked to changes in the statistical structure of the training data. Before augmentation, the number of samples across categories exhibited a distinct long-tail distribution; for example, “Tomato two-spotted spider mites leaf” had only two samples, whereas “Blueberry leaf” had 755 samples each. Using the ratio of the maximum to minimum number of samples per category as the Imbalance Ratio (IR), prior to augmentation,

$$IR_{\mathrm{before}}=\frac{755}{2}\approx 377.5.$$

After processing with CALM-Aug, the filling of tail categories became systematic, with the smallest category increasing to 54 and the largest to 5101. Correspondingly,

$$IR_{\mathrm{after}}=\frac{5101}{54}\approx 94.5.$$

When the *IR* converges, the “extremely sparse region” at the long tail shrinks, and the differences in class frequencies shift toward a range that is easier to learn.

Given the substantial increase in the total sample size after augmentation, a simple comparison of the original count variance and standard deviation would be obscured by the scale effect. Thus, we switched to using a logarithmic scale to describe the multiplicative dispersion of class frequencies. The standard deviation, calculated using the log(⋅) of the sample counts for each class, was approximately 1.20 before augmentation and 1.19 after. This value remains relatively stable as the scale expands; its slight decline indicates that, as the total volume increases, the fluctuations in category frequencies on a multiplicative scale have narrowed, and the distribution better aligns with a scenario that is “stably optimizable”.

Building on this, we can further characterize the direct contribution of CALM-Aug to training learnability using the “proportion of effective samples”. Here, we define “effective categories” as those with a sample size of at least $\;N_{0}$, and calculate the effective category coverage and the proportion of effective samples accordingly. Setting a threshold of $N_{0}=200$ (to exclude high-variance updates caused by extremely low-frequency categories), the number of categories meeting this threshold before augmentation was 17/30 (56.7%), which increased to 29/30 (96.7%) after augmentation; correspondingly, the proportion of samples from valid categories rose from 80.8% to 99.9%. This result implies that the vast majority of gradient updates during training originate from a set of classes with “sufficient sample size to support stable learning”, and long-tail classes are no longer in an extremely sparse range where it is difficult to form representations.

From the perspective of training dynamics, CALM-Aug, through lesion-level recombination, not only increases the probability of rare diseases appearing in each iteration—thereby alleviating the issue of dominant diseases dictating gradient directions—but also eliminates the model’s reliance on specific locations and single scales through the introduction of diverse backgrounds. Ultimately, detection accuracy achieves a stable improvement in both mAP50 and mAP50–95. Therefore, CALM-Aug is essentially a data distribution reconstruction module designed for the detection of long-tail agricultural diseases. It balances the training frequency of various disease types at the source, providing a healthier and more stable training foundation for subsequent fine-tuning of the model architecture.

### 2.3. Model-Level Optimization: YOLO11-ARL

After completing the data-level reconstruction of disease distribution, the model architecture becomes the key factor influencing detection performance. Plant disease detection tasks face numerous unique challenges: lesions are typically small in scale, and their texture and color differ only slightly from healthy leaf regions. Field backgrounds are complex and variable, containing interfering factors such as varying lighting, occlusion, and soil; simultaneously, rare disease samples are limited, placing higher demands on the model’s feature extraction capabilities. If the higher layers of the network are overly abstract, they are prone to losing detailed information about lesions; if the classification and localization tasks share too many parameters, gradient updates may interfere with each other, leading to localization errors for small-scale lesions; and if the regression loss is overly sensitive to minor shifts, training is prone to oscillation [11,12,13]. To address these challenges, we adopted YOLO11n as the base architecture. While maintaining the original backbone topology, we locally restructured the critical paths to tackle the aforementioned difficulties in plant disease detection, thereby constructing the YOLO11-ARL model.

YOLO11-ARL does not alter the overall Backbone–Neck–Head structure but introduces refined designs tailored to lesion features: at the feature representation level, it enhances the preservation of details for small-scale lesions; at the parameter allocation level, it optimizes the decoupling and collaboration between classification and localization tasks; and at the loss function level, it adjusts the tolerance for small-target offsets to ensure a smoother training process. Through these targeted interventions, YOLO11-ARL improves lesion detection accuracy while avoiding uncontrolled growth in model size, thereby better addressing the needs of disease identification in real-world agricultural scenarios.

#### 2.3.1. Overall Architecture of YOLO11-ARL

YOLO11-ARL is based on the YOLO11n architecture [14,15,16] and continues to use the three-stage Backbone–Neck–Head architecture. The Backbone handles feature extraction across multiple levels, while the Neck employs upsampling and concatenation to perform fusion operations across different scales [17,18]. The Head provides detection results at the three major scales of P3, P4, and P5; however, the network’s downsampling ratio and the resolution of the corresponding features remain unchanged, and the depth of the backbone has not been altered.

**Figure 2:**
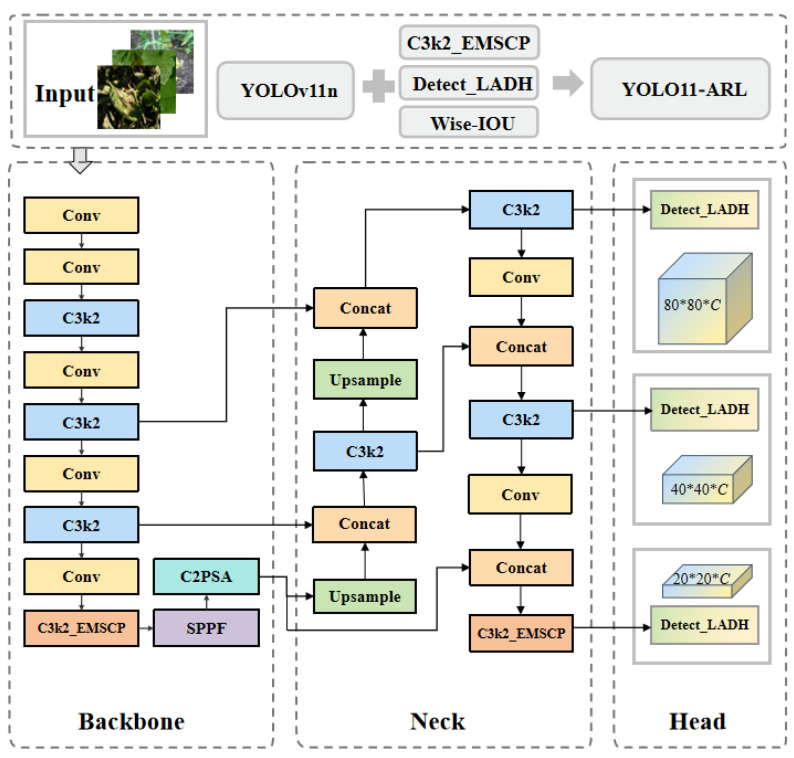
Schematic of the YOLOv11n-ARL model architecture.

Compared to the original architecture, the model incorporates three modifications. First, in the high-level semantic section of the backbone, the original C3k2 module is replaced with C3k2_EMSCP to enhance multi-scale semantic representation capabilities. A C3k2_EMSCP module is further inserted at the top-level semantic layer (P5) to improve deep-layer features, thereby addressing the issue where small-scale objects become progressively weaker in representation at deeper layers. In the detection output stage, the original Detect module is replaced with Detect_LADH, structurally separating the classification and regression paths. Additionally, Wise-IoU is adopted at the loss function layer to regulate the distribution of regression gradients. The overall architecture is shown in Figure 2.

All the modifications mentioned above occur in areas that significantly impact gradient propagation. The feature fusion path in the Neck remains unchanged, and the performance of the three output scales shows no variation; therefore, the model’s parameter size and computational complexity remain at a level close to that of the original model.

#### 2.3.2. EMSCP Module

Enhancing feature representational capacity can be achieved by incorporating Transformers, self-attention, or stacking additional convolutional layers [19]. However, the discriminative information in plant disease detection is primarily manifested in local-scale textures and comparative relationships between neighboring regions, without the need for long-range dependencies. Global attention structures often entail significant computational costs on high-resolution feature maps and can alter the statistical properties of convolutional features, thereby causing convergence stability issues in lightweight models.

**Figure 3:**
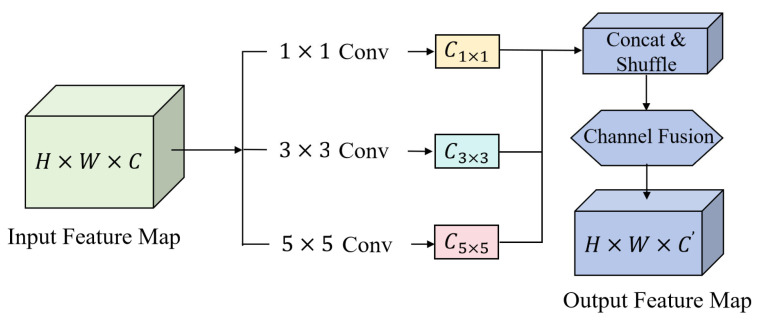
Schematic of the EMSCP architecture.

EMSCP utilizes parallel multi-scale convolutional branches for local context modeling [19,20]. It expands the effective receptive field while maintaining the consistency of the convolutional backbone; the architecture is shown in Figure 3.

Assuming the input feature size is C × H × W and the set of parallel convolution kernels is *kᵢ*, then the additional computational complexity can be approximately expressed as follows:

$$\Delta \mathrm{FLOPs}\approx \sum_{i}C^{2}k_{i}^{2}HW.$$

Its growth follows a linear scaling pattern with respect to the number of convolutions, rather than the complexity of global matrix multiplication [21]. For small-scale, low-contrast lesion targets, multi-scale local modeling can preserve essential details in high-level semantic spaces. Compared to simply increasing network depth, this architecture focuses on modifying the organization of scales to optimize feature discrimination.

#### 2.3.3. LADH Detection Head

The detection head must perform both classification and regression. When using a shared detection head, both tasks are updated within the same parameter subspace, denoted as shared parameters $\theta_{s}$, and their update gradients can be expressed as

$$g_{s}=\nabla_{\theta_{s}}L_{\mathrm{cls}}+\nabla_{\theta_{s}}L_{\mathrm{reg}}.$$

**Figure 4:**
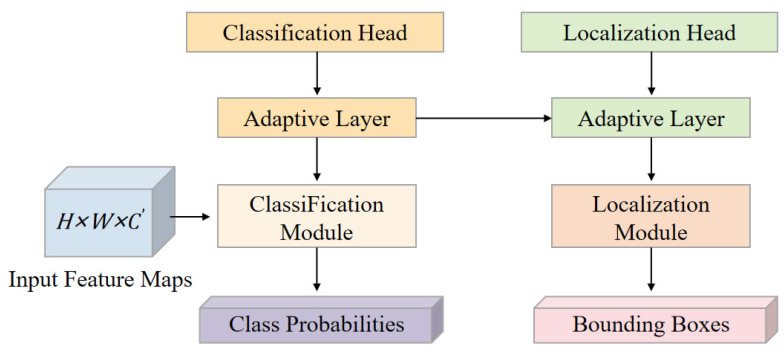
Schematic of the LADH detection head structure.

When the directions of the two gradients differ, the update process may become mutually constrained, leading to instability in training. To improve the expressive power of the detection head, methods such as incorporating attention weights or more complex branch structures can be employed [22,23]; however, these approaches often result in an increase in the number of parameters and introduce new uncertainties in optimization. LADH utilizes structural decoupling to separate the classification and regression paths, reducing the number of shared parameters. Its structure is shown in Figure 4.

The comparison results of different detection head architectures are shown in Table 2.

With a smaller parameter scale, LADH achieves better detection performance, indicating that structural decoupling can alleviate mutual interference between tasks.

#### 2.3.4. Wise-IoU Regression Optimization

Regarding the selection of regression loss functions, methods such as IoU, GIoU, DIoU, and CIoU improve the optimization objective by introducing geometric constraints. However, when dealing with small-sized targets, even minor shifts in the predicted bounding box can cause significant changes in the IoU value. If the loss function has a steep gradient in the low IoU range, it will result in excessive update steps during training, thereby affecting the stability of the convergence process.

The overall loss function is expressed as

$$L=L_{\mathrm{cls}}+\lambda L_{\mathrm{reg}},$$

and the corresponding gradient variance can be written as

$$Var(g)=Var(g_{\mathrm{cls}})+\lambda^{2}Var(g_{\mathrm{reg}})+2\lambda Cov(g_{\mathrm{cls}},g_{\mathrm{reg}}).$$

**Figure 5:**
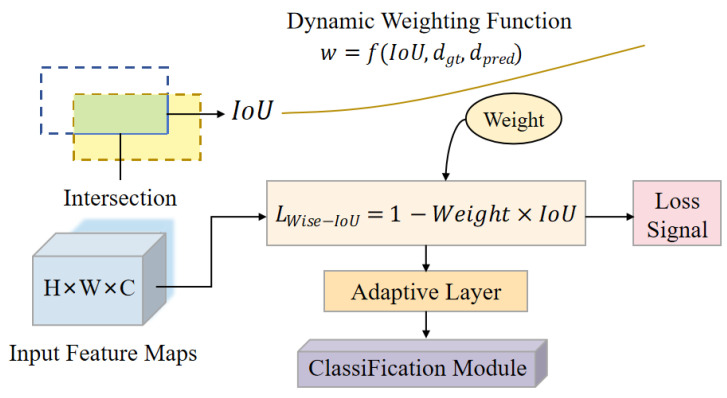
Schematic of the Wise-IoU regression algorithm.

Wise-IoU adjusts the gradients across different prediction quality intervals to make them smoother [24,25], thereby reducing $Var(g_{\mathrm{reg}})$ and mitigating fluctuations in the covariance with classification gradients. Compared to loss functions that simply sum geometric constraint terms, this approach focuses more on enhancing the overall stability of the process. Its position within the network is shown in Figure 5.

#### 2.3.5. Ablation Study and Efficiency Analysis

To evaluate the actual contributions of each component, we conducted both individual and combined ablation experiments. All models were trained using the same methodology, including an input size of 640 × 640, 100 training epochs, a batch size of 32, the SGD optimizer with cosine learning rate scheduling enabled, Mosaic enhancement disabled during the late training phase, and no early stopping mechanism. All experiments were conducted on an NVIDIA RTX 2080Ti GPU, with all training conditions kept consistent except for structural differences.

The results of the ablation experiments are shown in Table 3.

Table 3 shows that, while maintaining consistent training settings, stable changes in various metrics occur when performing individual structural adjustments to the model. After replacing the regression loss with Wise-IoU, mAP50 increased from 83.1% to 84.2%; after introducing EMSCP, mAP50 reached 84.3%. When using the LADH detection head, precision increased to 84.5%, and recall reached 79.2%. None of these three adjustments altered the network’s overall topology, yet they yielded sustained improvements across different performance metrics.

Performance improved further when combining the two modules: when EMSCP and LADH were applied simultaneously, precision reached 86.4% and mAP50 reached 84.3%; when Wise-IoU and LADH were combined, precision rose to 86.6%. Compared to using them individually, this growth demonstrated an additive effect, indicating that the effects of each approach are not mutually exclusive.

When all three modifications are incorporated, the model achieves its best performance in the current experimental setting, with a precision of 88.5% and a recall of 79.9%, and mAP50 reached 84.9%. Compared to the baseline, mAP50 improved by 1.8 percentage points, while the number of parameters decreased from 2.588 M to 2.242 M, and the weight file size shrank from 5.2 MB to 4.6 MB. GFLOPs remained roughly at the same level, and inference speed remained within the range of lightweight models.

These results indicate that the performance improvements did not rely on an increase in model size but rather stemmed largely from changes to the structural architecture itself. The enhanced high-level representation optimized the discriminative power of semantic features, the separation of detection heads reduced cross-contamination between classification and regression, and the smoothing of the regression loss made gradient updates more stable. Achieving higher detection accuracy while maintaining a similar model size makes this architecture particularly relevant for application in resource-constrained agricultural environments.

### 2.4. Model Compression via LAFDP

After CALM-Aug completed training distribution reshaping and YOLO11-ARL completed key path structural optimization, the model already demonstrated relatively stable detection performance on the PlantDoc dataset. However, practical agricultural applications typically target drone inspection platforms, mobile devices, and embedded systems rather than high-performance servers; therefore, model size, computational complexity, and inference latency still require further reduction. Under these circumstances, reducing model redundancy without compromising the key lesion representation pathways has become a critical challenge for achieving edge deployment.

To address this, this paper introduces a Layer-wise Adaptive Feature-guided Distillation Pruning (LAFDP) method based on YOLO11-ARL, and names the compressed model YOLO11-ARL-PD. LAFDP does not alter the model’s backbone topology; instead, it removes redundant computations through structured channel pruning and restores the compressed model’s expressive power via distillation fine-tuning under the constraints of a teacher model. Unlike methods that directly reduce network width or simply replace components with lightweight models, this approach emphasizes preserving key feature pathways closely related to lesion detection within a given compression budget, thereby balancing detection accuracy with deployment efficiency.

#### 2.4.1. Hierarchical Adaptive Channel Pruning

The first stage of LAFDP is hierarchical adaptive channel pruning. The core of hierarchical adaptive pruning lies in overcoming the shortcomings of fixed-ratio pruning. While uniform-ratio pruning is easy to implement, it often assumes that the degree of redundancy is consistent across all layers, making it difficult to reflect the actual differences in the roles of different layers in feature representation and detection decisions. For plant disease detection tasks, shallow layers handle texture and edge feature extraction, intermediate layers manage feature fusion, and deep layers directly influence classification and localization outputs. If fixed-ratio compression is applied, it can easily lead to information loss in critical layers and insufficient compression in redundant layers, thereby degrading model performance. To address this, this paper allows the retention level of channels in each layer to vary according to differences in structural redundancy and task sensitivity, while maintaining a unified compression objective, to enhance the rationality of the compression process. This paper first inputs the uncompressed YOLO11-ARL model into a structured compression module to perform channel importance ranking and pruning under a global pruning scheme. Simultaneously, the overall compression intensity is controlled by setting a target acceleration ratio, and the stability of the compression process is enhanced through sparse training-related settings. The compression magnitude is governed by a global budget rather than by manually setting a fixed pruning ratio for each layer individually.

This strategy is adopted because plant disease detection tasks exhibit significant differences in their reliance on features at various levels. Shallow-layer features are primarily responsible for extracting leaf texture, edge contours, and local lesion details; they are highly sensitive to information loss, and excessive compression can easily compromise fundamental representations. Mid-level features are more prone to accumulating redundant representations during multiscale fusion and semantic integration, and thus typically offer greater compression potential, whereas deep layers and the detection head near the output directly participate in classification and localization decisions, making their structural stability critical to the final detection results. While applying uniform pruning at a fixed ratio is straightforward, it risks overlooking the functional differences among layers in the detection task, potentially leading to over-compression of feature pathways crucial for lesion identification.

**Figure 6:**
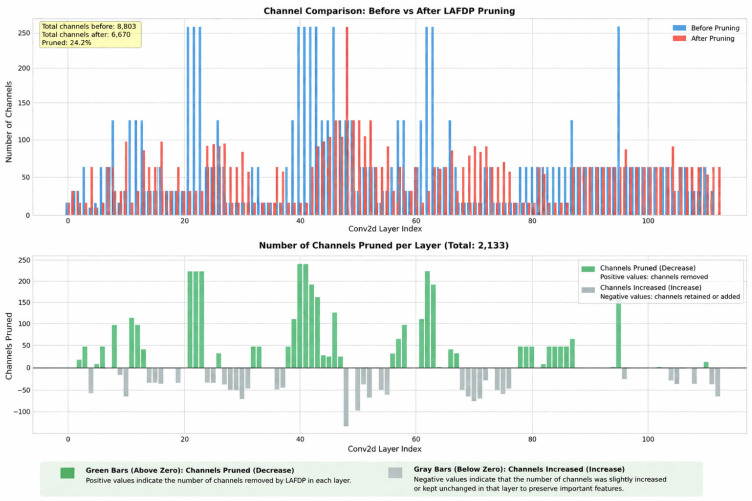
Visualization of channel distribution in the YOLO11-ARL model before and after LAFDP.

Based on the above considerations, this paper does not adopt a simple compression method of “equal-ratio pruning across all layers”, but instead performs global structured pruning under a unified compression objective, enabling each layer to exhibit differentiated channel retention results based on its actual redundancy. This differentiation is not artificially imposed but rather a natural outcome of the interaction between the network’s internal redundancy distribution, structural coupling relationships, and the overall compression budget. Therefore, “hierarchical adaptivity” essentially manifests as follows: the compression process does not aim for uniform reduction across all layers but prioritizes retaining key feature paths that are more sensitive to lesion texture, boundary information, and detection decisions, while ensuring the overall compression rate as much as possible. The changes in channels across each layer before and after pruning are shown in Figure 6.

The total number of channels in the model decreased from 8803 to 6670, a reduction of 2133 channels, resulting in a channel compression rate of approximately 24.2%. This compression process did not occur uniformly across all layers but exhibited clear inter-layer differences: channel reduction was most significant in the middle layers, with some layers losing over 200 channels; in contrast, channel changes were notably more gradual in the shallow layers near the input and the deep layers near the detection output.

This result clearly demonstrates that the compression process in LAFDP is not a simple fixed-ratio pruning. If uniform pruning were applied, the channel reduction trends across different layers would typically follow a more linear distribution, and such pronounced differences in compression intensity between layers would not be observed. However, the results shown in Figure 6 indicate that, under a unified compression budget constraint, different layers exhibit varying compression intensities based on their actual redundancy levels and task sensitivity. The intermediate layers perform a significant amount of feature combination and semantic fusion, making them more prone to redundant representations and thus offering greater potential for compression; the shallow layers are responsible for extracting basic details such as leaf edges and lesion textures, requiring higher integrity in early-stage representations, and are therefore compressed more conservatively. The deep layers and the detection head directly participate in classification and localization outputs, and their structural stability is more closely related to the final prediction quality, so they are similarly preserved with greater caution. It can thus be seen that what Figure 6 illustrates is not a manually preset “fixed hierarchical ratio”, but rather a result of hierarchical differentiation automatically formed under the unified compression objective.

Furthermore, in some layers shown in the figure, channel changes are not entirely monotonic, and even local negative values may appear. This does not imply instability in the compression process or statistical errors, but is primarily related to channel reorganization performed during pruning to maintain network structural connectivity and feature transmission continuity. LAFDP does not perform mechanical pruning on the network but simultaneously considers structural feasibility, path integrity, and feature expression continuity during the compression process. For this reason, although certain layers participate in the compression process overall, their local structures may exhibit non-monotonic changes due to path matching or module reconstruction.

From the perspective of the detection task, this outcome is clearly justified. Plant disease detection requires not only the identification of category differences but also sufficient sensitivity to local anomalies within small lesions, blurred boundaries, and complex backgrounds. If the compression strategy fails to distinguish the functional differences between layers, it may reduce the model size while simultaneously compromising the ability to express key pathological features. The hierarchical differences shown in Figure 6 demonstrate that LAFDP removes as many redundant channels as possible within a unified compression budget, while prioritizing the retention of key feature paths that are more sensitive to lesion texture, edge information, and detection decisions. This preserves a stable structural foundation for the subsequent distillation recovery stage and provides visual support for the methodological design of “Hierarchical Adaptive Channel Pruning” proposed in this paper.

Building on this, this paper further introduces distillation-based fine-tuning under teacher model constraints to restore the expressive power of the pruned model in a lower-dimensional parameter space.

#### 2.4.2. Distillation-Driven Semantic Reconstruction

Channel compression reduces the model’s degrees of freedom but may also diminish its expressive power. To regain the original discriminative capability in a lower-dimensional feature space, this paper incorporates distillation training [26] after pruning, enabling the student model to align with the teacher model in terms of semantic structure.

**Figure 7:**
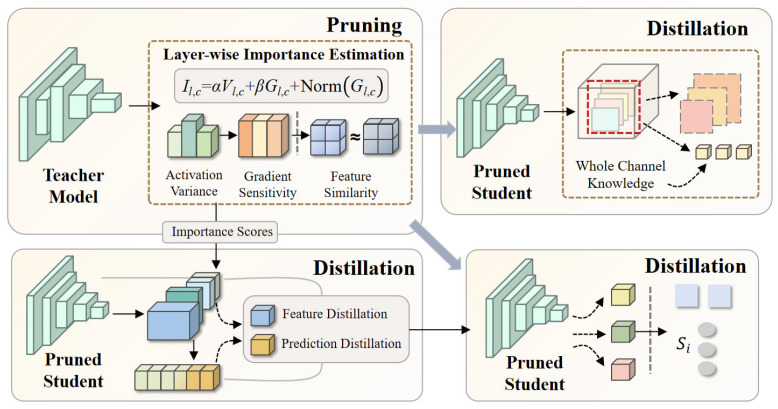
Schematic of the distillation architecture.

The distillation workflow is shown in Figure 7.

During training, while the student model maintains its own detection task loss, prediction distribution distillation and feature distillation are incorporated under the constraint [27]. The KL divergence is used to align the student’s output with the teacher’s probability distribution, and alignment operations are performed on the corresponding scale feature maps, enabling the student to reconstruct high-level semantic representations in the compressed space. The entire loss function can be expressed as

$$L=L_{\mathrm{task}}+\lambda_{1}L_{\mathrm{pred}}+\lambda_{2}L_{\mathrm{feat}}$$

where $\;L_{\mathrm{task}}$ includes classification loss, regression loss, and DFL. The distillation process aligns with the pruning dimension; pruning occurs at the channel dimension, and distillation achieves overall alignment at the probability distribution level of the channels, rather than matching point-by-point. This consistency makes compression and reconstruction continuous processes within the same structural path, rather than two independent stages.

#### 2.4.3. Performance of the Compressed Model

Under identical training conditions, we applied LAFDP compression and distillation to the YOLO11-ARL model described earlier, resulting in YOLO11-ARL-PD. Its performance is shown in Table 4.

As shown in Table 4, after LAFDP processing, the number of model parameters decreased from 2.242 M to 1.244 M, a reduction of 44.51%; FLOPs decreased from 5.243 G to 4.018 G, a reduction of 23.37 percentage points; model size decreased from 4.6 MB to 2.8 MB; and inference speed increased to 1.30 times that of the original model. Meanwhile, model accuracy did not decrease; precision, recall, and mAP@0.5 improved from 88.5%, 79.9%, and 84.9% to 89.0%, 80.5%, and 85.3%, respectively. This indicates that the compression process mainly removed redundant channels, while the key feature expression paths related to lesion detection were effectively preserved. In summary, LAFDP significantly reduced model complexity without destroying the key structural pathways upon which lesion detection depends, demonstrating that the current compression process achieves a good balance between lightweight design and accuracy preservation.

To further validate the effectiveness of LAFDP, this paper compares LAFDP with several representative pruning methods—including LAMP, Group-Taylor, Group-Hessian, and Slim—under the same distillation and restoration settings. To ensure a fair comparison, all methods started from the same baseline model, underwent restoration training under the same distillation strategy, and maintained the GFLOPs of the compressed models at the same level (4.0 G). Under these conditions, we compared the differences in detection performance, parameter scale, and model size among various pruning strategies to evaluate the comprehensive advantages of LAFDP in lightweight plant disease detection tasks.

As shown in Table 5, LAFDP achieved the best results across all three metrics—precision, recall, and mAP50—while also achieving the smallest number of parameters and model size. This result indicates that the advantage of LAFDP lies not only in its compression ratio but also in the degree of alignment between the compressed structure and the distilled reconstruction phase. For plant disease detection tasks, the model must retain sensitivity to the texture of minute lesions, edge contours, and differences in complex backgrounds. If the pruning process causes excessive damage to critical feature pathways, it is difficult to fully compensate for the loss in accuracy even with a unified distillation recovery strategy. The results in Table 5 indicate that, under the same distillation constraints, the LAFDP-compressed model achieves the best restoration performance. This implies a higher consistency between its pruning results and the subsequent distillation stage, thereby indirectly validating the method’s applicability in lightweight disease detection scenarios.

Combining Table 4 and Table 5 reveals that LAFDP’s performance advantage does not stem from model scale expansion, but rather from the synergistic effect of structured compression and distillation-based restoration. On one hand, structured pruning effectively reduces the number of parameters, FLOPs, and model size; on the other hand, distillation-based restoration under the constraints of the teacher model helps the compressed model re-establish effective representations within a more compact parameter space, thereby maintaining high detection performance even under lightweight conditions. Thus, LAFDP plays the role of “capacity reconstruction” within the overall framework of this paper. It further transforms the distribution optimization of CALM-Aug and the structural optimization of YOLO11-ARL into deployable lightweight models, enabling the final method to achieve an optimal balance between detection accuracy, inference efficiency, and deployment feasibility.

#### 2.4.4. Balance Between Structural Capacity and Performance

Experimental results indicate that reducing model capacity does not result in accuracy loss but rather leads to a slight improvement. This suggests that YOLO11-ARL is somewhat overparameterized in its uncompressed form. Channel pruning reduces redundant degrees of freedom, making the representation more compact, while distillation constraints realign the decision structure within the compressed space—much like applying a form of structural regularization to the student model.

In agricultural disease detection tasks, where the number of samples is limited and there are subtle differences between categories, excessive model capacity does not necessarily enhance generalization ability. LAFDP controls capacity while ensuring semantic consistency, making the model’s complexity better suited to the dataset size, thereby achieving compression while maintaining or even optimizing detection performance.

Within the overall architecture of this paper, CALM-Aug handles the task of distribution reconstruction, YOLO11-ARL focuses on improving the critical path, and LAFDP performs capacity reconstruction. These three methods create a progressive improvement relationship across the three dimensions of data, structure, and capacity, thereby enabling the final model to achieve a relatively stable equilibrium in terms of accuracy, efficiency, and deployability.

## 3. Results and Discussion

### 3.1. Comparative Experimental Analysis

After standardizing the training settings, the final model was compared with mainstream detection algorithms on the PlantDoc dataset, as shown in Table 6 below.

Regarding mAP@0.5, YOLO11-ARL-PD achieved 85.3%, a 2.2 percentage point improvement over YOLOv11n. When using the stricter mAP@0.5–0.95 threshold, the gap widened by another 2.8 percentage points, and both precision and recall increased, without creating the typical trade-off between accuracy and recall.

When examining the statistics before and after pruning and distillation in terms of model complexity, we can see that the number of parameters has been reduced from 2.242 M to 1.244 M, and FLOPs decreased from 5.243 G to 4.018 G. Despite the reduced capacity, mAP50 improved from 84.9% to 85.3%, with both precision and recall increasing. This trend indicates that the performance improvement stems not from an expansion of the model’s scale but from the reconstruction of structural paths and the reallocation of capacity.

Achieving higher accuracy while maintaining similar computational complexity suggests that the performance gap stems from differences in feature representation efficiency rather than differences in network depth or width.

### 3.2. Validation of Cross-Dataset Generalization Capabilities

To assess whether the model can operate normally when faced with different data sources, we specifically selected several plant disease datasets publicly available on Roboflow and Kaggle for separate testing. These datasets differ from PlantDoc in terms of acquisition settings, imaging equipment, background complexity, and annotation methods, thereby allowing us to evaluate the model’s detection stability under varying distribution conditions.

During data preparation, only plant diseases with exactly the same semantic categories as those in PlantDoc were selected for evaluation; categories with similar names but differing scopes were excluded. This minimizes uncertainties caused by category mapping. Additionally, performed duplicate checks between external data and training data to prevent sample overlap from interfering with test results.

After training on the PlantDoc training set, each model was directly applied to the external dataset for inference and evaluation without any additional adjustments. Parameters such as input resolution, confidence, and NMS were kept constant to ensure the fairness of the comparison. Using mAP@0.5 as one of the evaluation metrics, as shown in Table 7.

In cross-dataset testing, the improved model achieved significant improvements in the vast majority of overlapping categories, particularly demonstrating greater stability in detection results for samples with weak textures and complex backgrounds. Compared to the baseline model, the prediction boxes for some categories showed higher overlap with lesion areas, and both false positives and false negatives were reduced. However, the magnitude of improvement varies across different categories, with a few categories still exhibiting low accuracy or limited improvement. This phenomenon is closely related to factors such as differences in shooting environments across datasets, the distinguishability of the disease symptoms themselves, and inconsistencies in annotation boundary standards. In particular, categories such as “Tomato leaf yellow virus”, which feature diffuse symptom distribution and unclear local boundaries, better illustrate the challenges of fine-grained disease identification in complex agricultural scenarios [28]. Such diseases typically manifest as overall yellowing, color gradients, and localized textural degradation, lacking distinct, well-defined lesion boundaries. Consequently, they are more easily confused with phenomena such as changes in lighting, nutrient deficiencies, or natural leaf senescence during detection tasks, leading to relatively low recognition accuracy. Future work could explore the introduction of uncertainty quantification mechanisms, such as multi-stream feature fusion, to calibrate detection evidence for ambiguous targets, thereby reducing the model’s classification bias on samples with weak features [29].

Combining the results of data augmentation and structural optimization discussed earlier reveals that the feature representations generated by our method are not limited to a single data distribution but acquire stronger cross-scenario adaptability during training. CALM-Aug expands the coverage of the sample distribution through category-aware augmentation and a teacher-guided filtering mechanism. This aligns with the masked contrastive learning concept proposed by MaskCon, which reinforces the model’s ability to decouple features under complex distributions by enhancing the consistency constraints of augmented samples [30]. Although previous studies have combined architectures such as Mamba with YOLO [31,32,33], these methods often rely on complex super-resolution reconstruction or incur significant computational overhead. In contrast, YOLO11-ARL-PD maintains a lightweight architecture while further enhancing the model’s ability to characterize complex backgrounds and lesions with weak textures. Consequently, the model can generally maintain a stable recognition performance even when imaging conditions and distribution shifts occur in external datasets. This indicates that our method is not only effective on the PlantDoc dataset but also possesses potential for cross-dataset generalization.

### 3.3. Visual Analysis of Object Detection

To further analyze the model’s varying responses at the spatial level, we adopted the HiResCAM method to visualize features from the higher layers of the detection network [34,35]. Unlike Grad-CAM, which relies on global average pooling to determine the weights of each channel, HiResCAM directly utilizes pixel-wise gradient information when generating response maps, thereby preserving higher-resolution spatial structures.

**Figure 8:**
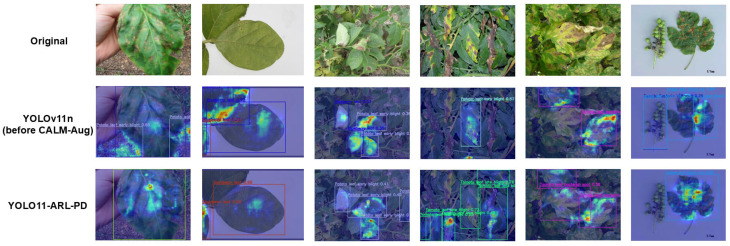
Heatmap of leaf detection results based on HiResCAM.

Let the feature map of the k th feature channel be $A^{k}$ and the corresponding class score be y. The response map of HiResCAM can be expressed as:

$$L_{\mathrm{HiResCAM}}=\mathrm{ReLU}\left(\sum_{k}\frac{\partial y}{\partial A^{k}}⊙A^{k}\right)$$

where ⊙ represents the element-wise product. Unlike the channel-weighted average form, this expression directly utilizes the spatial distribution of gradients. This allows the response map to maintain a high level of resolution at boundaries and for small-scale targets. Since no additional spatial compression is applied, the heatmap can more accurately represent the specific spatial information on which the model’s decision-making relies [36]. The visualization results produced by HiResCAM are shown in Figure 8.

For single-target cases, both models can detect the lesion, though their heatmap distributions differ. In some samples, the baseline model’s response area extends into adjacent leaf texture regions, whereas the improved model’s response is more concentrated in the center of the lesion, with clearer gradient responses at the edges and greater overlap between the prediction bounding box and high-response areas.

For samples with lesions only at the leaf margins, the baseline model’s response attenuates at the leaf edges, and some samples exhibit missed detection at the margins. In contrast, the improved model’s heatmap maintains a continuous distribution along the lesion contours, establishing a stable response even in partially visible regions. Such samples demand higher spatial resolution and gradient stability, and the improved model demonstrates greater robustness under these conditions.

In scenarios with densely clustered lesions, where lesions are closely distributed, the baseline model’s thermal response tends to blend across adjacent regions, resulting in some prediction boxes containing multiple targets. In contrast, the improved model’s response map exhibits a more distinct local separation structure, with clearer boundaries between high-response zones across different lesion areas, leading to greater separation in the corresponding prediction boxes.

In occlusion scenarios, where lesions are partially obscured by leaf veins or other structures, the baseline model exhibits discontinuous responses to such samples, and prediction boxes occasionally shift. In contrast, the improved model forms a continuous high-response region even in visible areas; even when the target is only partially exposed, its response remains stable, and the prediction boxes are more concentrated on the relevant regions.

As shown in the HiResCAM visualizations, the improved model exhibits a more compact and distinct spatial response distribution. The alignment with individual target boundaries has been enhanced, resulting in fewer missed detections of targets at the edges. Densely arranged targets are separated more reliably, and obscured targets still elicit a response. The thermal distribution aligns with the trends observed in quantitative metrics, demonstrating that the performance improvements are reflected in the spatial domain.

## 4. Conclusions

This paper addresses the challenges of long-tail distribution of plant disease categories, small-scale targets, and complex background interference. By optimizing YOLOv11 across three layers—data, architecture, and capacity—the experimental results demonstrate a consistent improvement in detection performance while maintaining a lightweight model.

From a data perspective, CALM-Aug reshapes the distribution of training data. Without modifying the network architecture, it significantly improves detection metrics: mAP@0.5 increased from 58.7% to 83.1% and mAP@0.5–0.95 reached 68.4%. This change indicates that the distribution of training data directly influences the model’s decision boundaries.

Structurally, after fine-tuning the critical paths via EMSCP, LADH, and Wise-IoU, the model achieves further performance improvements without expanding its parameter capacity. When these three modules are integrated, the mAP@0.5 reaches 84.9%, and precision rises to 88.5%. The improvements stem from more stable boundary regression and enhanced multi-scale representation capabilities.

In terms of model size, the incorporation of LAFDP for structured pruning and distillation reconstruction reduces the number of parameters from 2.242 M to 1.244 M. FLOPs were reduced by 23.37%, and inference speed increased by approximately 30%. Despite the reduction in capacity, mAP@0.5 further increases to 85.3%. The compression process did not compromise key performance pathways; semantic coherence was maintained even after removing redundant channels.

Benchmarking results show that YOLO11-ARL-PD achieves 89.0% precision and 71.2% mAP@0.5–0.95, outperforming many mainstream lightweight detection models. Cross-crop testing demonstrates that the improved model consistently performs better on public datasets for tomato, corn, and apple. Visual results indicate that the model exhibits more focused responses and better boundary alignment in scenarios involving isolated objects, objects with visible edges, densely clustered objects, and occluded objects.

Overall, the experimental results demonstrate a consistent trend of improvement across three dimensions: numerical metrics, class generalization, and spatial response. This indicates that the performance gains achieved by the proposed method do not rely on expanding the model’s size but are instead realized through a combination of data distribution reconstruction, key structural optimization, and capacity reconfiguration. Under lightweight constraints, the model still maintains a relatively stable detection performance, suggesting it has potential for deployment in real-world agricultural scenarios.

However, the validation in this paper is primarily based on public datasets and offline testing environments, and field deployment evaluations on existing agricultural drone platforms have not yet been completed. Therefore, future work will focus on conducting further research in real-world flight scenarios. First, the model will be deployed onto existing agricultural drones or onboard edge computing platforms to test real-time inference capabilities, stability, and resource consumption during actual field inspection processes. Second, we will focus on evaluating the model’s recognition performance under external noise disturbances such as drone vibrations, changes in flight attitude, motion blur, image defocusing, sudden changes in lighting, and complex weather conditions, to more comprehensively validate its engineering applicability. To address these field noise characteristics, we may introduce enhancement strategies for blur degradation and dynamic disturbances, robust feature modeling methods, or temporal information constraint mechanisms to improve the model’s stable detection capabilities under continuous flight data collection conditions. Subsequently, we will conduct more systematic validation of the model’s long-term generalization and cross-domain adaptability using larger-scale, cross-regional, and cross-seasonal datasets, thereby providing more robust experimental evidence for practical implementation in smart crop protection scenarios.

## Figures and Tables

**Table 1:** Comparison of detection performance across different augmentation strategies.

Metric	Precision/%	Recall/%	mAP50/%	mAP50–95/%
No augmentation	55.6	55.1	58.7	41.5
Standard copy-paste	66.1	62.0	67.0	50.5
Class-aware copy-paste	71.1	71.9	67.4	52.1
CALM-Aug without teacher screening	66.7	64.5	68.0	52.5
CALM-Aug (full)	82.6	78.1	83.1	68.4

**Table 2:** Detection head comparison.

Models	Precision/%	Recall/%	mAP50/%	Parameters	GFLOPs
YOLO11-LSCD	82.5	78.5	83.1	2,422,377	5.6
YOLO11-MultiSEAMHead	83.4	77.2	83.2	4,600,162	6.1
YOLO11-SEAMHead	84.1	76.3	83.6	2,496,226	5.8
YOLO11-RSCD	84.3	77.6	83.4	2,422,121	5.6
YOLO11-EfficientHead	83.3	77.8	83.5	2,325,218	5.2
YOLO11-LADH	84.5	79.2	84.1	2,287,202	5.2

**Table 3:** Ablation study of YOLO11-ARL.

Test No.	Base Model	Wise-IoU	EMSCP	LADH	Precision P/%	Recall R/%	mAP50/%	Weights/MB	Parameters	FPS
1	YOLOv11n	×	×	×	82.6	78.1	83.1	5.2	2,588,002	129.9
2		√	×	×	84.1	78.9	84.2	5.2	2,588,002	129.9
3		×	√	×	83.9	78.4	84.3	5.2	2,543,202	104.6
4		×	×	√	84.5	79.2	84.1	4.7	2,287,202	121.9
5		√	√	×	85.9	77.8	83.7	5.2	2,543,202	104.6
6		×	√	√	86.4	77.9	84.3	4.6	2,242,402	103.3
7		√	×	√	86.6	76.8	83.4	4.7	2,287,202	121.9
8		√	√	√	88.5	79.9	84.9	4.6	2,242,402	103.3

Note: √ indicates the algorithm is used; × indicates the algorithm is not used.

**Table 4:** Performance comparison of YOLO11-ARL before and after LAFDP (pruning–distillation).

Metric	Before Pruning	After Pruning–Distillation	Absolute Change	Relative Change
FLOPs (G)	5.243	4.018	−1.225	−23.37%
Parameters (M)	2.242	1.244	−0.998	−44.51%
Model Size (MB)	4.6	2.8	−1.8	−39.13%
Inference Speed	1.00× (baseline)	1.30×	+0.30×	+30.0%
Precision (%)	88.5	89.0	+0.5	+0.56%
Recall (%)	79.9	80.5	+0.6	+0.75%
mAP50 (%)	84.9	85.3	+0.4	+0.47%

**Table 5:** Performance comparison of different pruning methods under identical distillation–reconstruction settings.

Method	Precision (%)	Recall (%)	mAP50 (%)	Parameters (M)	Model Size (MB)
LAMP	86.2	77.8	83.8	1.258	2.9
Group-Taylor	84.9	80.2	84.1	1.675	3.7
Group-Hessian	85.4	79.7	84.6	1.693	3.7
Slim	88.2	79.8	84.3	1.783	3.9
LAFDP	89.0	80.5	85.3	1.244	2.8

**Table 6:** Comparative experiment results.

Models	Precision/%	Recall/%	mAP@0.5/%	mAP@0.5~0.95/%
SSD	78.2	65.3	72.5	48.2
Faster-RCNN	82.1	70.4	76.8	53.1
Retinanet	81.5	68.7	75.9	51.8
EfficientDet	80.8	72.1	77.4	55.6
MobileNet-SSD	72.6	58.9	65.3	38.7
YOLOv5n	79.4	68.5	74.2	50.3
YOLOv5s	82.3	74.1	79.5	58.4
YOLOv7-tiny	80.9	73.6	78.8	56.9
YOLOv8n	81.7	72.8	78.1	55.2
YOLOv10n	82.9	75.4	80.6	60.5
YOLOv11n	82.6	78.1	83.1	68.4
YOLOv13n	84.2	77.6	82.7	67.1
YOLOv26n	84.3	72.3	80.8	67.4
WD-YOLO	60.4	60.1	65.4	53.1
E-TomatoDet	53.8	50.8	51.8	35.4
YOLO11-ARL-PD	89.0	80.5	85.3	71.2

**Table 7:** Comparison of generalization results across different datasets.

Dataset	Class	YOLOv11n	YOLO11-ARL-PD
Tomato Leaf Disease	Tomato early blight leaf	84.5	96.5
	Tomato Septoria leaf spot	65.2	70.8
	Tomato leaf	76.7	87.7
	Tomato leaf bacterial spot	86.6	92.6
	Tomato leaf late blight	76.8	89.2
	Tomato leaf mosaic virus	90.6	95.0
	Tomato leaf yellow virus	40.3	50.5
	Tomato mold leaf	94.3	91.5
Corn Leaf Disease	Corn gray leaf spot	72.2	77.3
	Corn leaf blight	75.7	85.5
	Corn rust leaf	68.1	75.9
Apple Leaf Disease	Apple scab leaf	88.0	91.5
	Apple leaf	94.4	97.0
	Apple rust leaf	89.1	94.2

## Data Availability

The dataset used in this study is the publicly available dataset PlantDoc.
